# Macroporous Poly(hydroxyethyl methacrylate) Hydrogels as Removable Antibiotic-Capture Liners for Endotracheal Tubes Designed to Prevent Ventilator-Associated Pneumonia

**DOI:** 10.3390/polym18172143

**Published:** 2026-09-02

**Authors:** David S. Jones, Roisin McCrory, Shu Li, Jordan Wilson, Gavin P. Andrews

**Affiliations:** School of Pharmacy, Queen’s University Belfast, 97, Lisburn Road, Belfast BT9 7BL, UKs.li@qub.ac.uk (S.L.); jordan.wilson@qub.ac.uk (J.W.); g.andrews@qub.ac.uk (G.P.A.)

**Keywords:** biomaterials, ventilator-associated pneumonia, hydrogel, nebulised gentamicin

## Abstract

Ventilator-associated pneumonia (VAP) is a life-threatening condition most frequently associated with the use of endotracheal tubes (ETs). This study describes the development of macroporous p(HEMA) hydrogels as removable antimicrobial liners for ETs to help prevent VAP. Hydrogels were prepared by free-radical polymerisation using varying sodium chloride concentrations and 40% or 60% *w*/*w* water. Increasing NaCl concentration enhanced porosity but reduced mechanical strength and improved swelling and gentamicin loading of hydrogels containing 60% *w*/*w* but not 40% *w*/*w* water. Gentamicin release was more controlled at lower NaCl concentrations, whereas hydrogels with higher porosity promoted rapid release. The formulation containing 60% *w*/*w* water and 0.1 M NaCl provided the best balance of mechanical integrity, drug uptake, sustained antimicrobial activity, and reduced bacterial adherence. Nebulisation studies showed that gentamicin-loaded hydrogels eradicated adherent bacteria and prevented recovery of viable organisms after repeated bacterial challenges. These findings indicate that macroporous p(HEMA) hydrogel liners offer a promising strategy for reducing endotracheal tube-associated infection.

## 1. Introduction

Since their original description by Wichterle and Lim [1], hydrogels have been widely used for pharmaceutical and biomedical applications, including drug delivery systems and medical devices (notably for tissue engineering, wound healing, and ocular and catheter-coating applications [2,3,4,5,6,7,8,9,10,11,12]). Poly(hydroxyethyl methacrylate) (p(HEMA)) and its copolymers have been extensively used as soft contact lenses, and their properties can additionally be modified to enable efficient drug delivery, to enhance comfort, and to reduce infection [13,14,15]. Hydrogels are also widely used as lubricious coatings for urinary catheters to facilitate catheter insertion and removal and to minimise damage to the urethral mucosa [16,17,18,19,20]. Hydrogels are commonly characterised as hydrophilic, 3-D polymer networks that can absorb large volumes of aqueous fluids whilst maintaining their mechanical structure [21,22,23,24,25]. Importantly, their mechanical and swelling properties may be engineered through choice and concentration of crosslinker and the incorporation of co-monomers [21].

Whilst hydrogels have been shown to be biocompatible biomaterials, their use as medical devices/implants is associated with certain limitations. For example, they exhibit intrinsically poor mechanical properties, which primarily limit their use to device coatings [24,25,26]. The pore size of hydrogels limits their use in both tissue engineering applications and drug delivery systems. Moreover, pore size directly affects the drug loading of hydrogels (using equilibrium swelling methods) and the subsequent drug release. For example, the authors illustrated this issue with p(HEMA) and p(hydroxyethyl methacrylate-co-methacrylic acid) (p(HEMA-co-MAA)) hydrogels and gentamicin [24]. The pore size of conventional hydrogels has also been cited as a barrier to cell survival and tissue regeneration when used in tissue engineering applications [27]. Given the importance of pore size, hydrogels have been prepared in which there are large interconnected pores that allow greater fluid and oxygen transport, termed microporous hydrogels [27]. Macroporous hydrogels have been successfully used for the delivery of therapeutic agents, e.g., ranitidine [28], adriamycin hydrochloride [29], and macromolecules, e.g., vascular endothelial growth factor and fibroblast growth factor [30].

In this study we propose that macroporous hydrogels can be used as liners of endotracheal tubes when used in conjunction with nebulised gentamicin for the prevention of ventilator-associated pneumonia (VAP). VAP is associated with increased stay within the intensive care unit (ICU), higher healthcare costs, and elevated mortality [31]. Previously we reported that, when administered by nebulisation, a high concentration of gentamicin in the lumen of the endotracheal tube was effective at preventing microbial biofilm formation on this surface [32]. It is proposed that the hydrogel liner described in this study be inserted into the lumen of the endotracheal tube and used to rapidly and effectively collect and concentrate nebulised gentamicin, thereby acting as an antimicrobial sink. As required, the liner can be removed and a replacement inserted without removing the endotracheal tube and inserting a replacement, a process normally associated with several oropharyngeal complications [33]. Gentamicin was chosen as the preferred antibiotic due its successful use as a nebulised solution for the treatment of acute lung conditions, cystic fibrosis, and non-cystic fibrosis bronchiectasis [34,35,36].

## 2. Materials and Methods

### 2.1. Materials

The sources of the materials used in this study have been previously described by the authors [22,24]. Gentamicin sulphate USP and Tris base (Tris(hydroxymethyl)aminomethane) were supplied by Taresh Chemicals (Banbridge, UK) and Melford Laboratories Ltd. (Suffolk, UK). 2-Hydroxyethylmethacrylate, ethylene glycol dimethacrylate, acetonitrile E CHROMASOLV for HPLC, 2-4,dinitrofluorobenzene, and sodium chloride were purchased from Sigma-Aldrich Co Ltd. (Dorset, UK). Muller Hinton Agar (MHA) and Muller Hinton Broth (MHB) were purchased from Oxoid Limited (Basingstoke, UK).

### 2.2. Bacterial Isolates

Two bacterial isolates were used in this study (*Staphylococcus aureus* and *Pseudomonas aeruginosa*), which were obtained from microbial biofilms on the surfaces of retrieved PVC endotracheal tubes from the ICU at The Royal Victoria Hospital, Belfast. Storage and maintenance of the isolates have been previously described by the authors [37,38,39].

### 2.3. Bacterial Growth Conditions

Bacteria were grown to the stationary phase by inoculating Mueller–Hinton broth (Oxoid Ltd., Basingstoke, UK) and incubating in an atmosphere of either air or 5% *v*/*v* carbon dioxide for 18 h. Following this, the respective bacterial cultures were centrifuged at 3000× *g* for 10 min and washed three times in sterile peptone water. The bacterial pellets were resuspended in Tris buffer (100 mM, pH 7.4) to a viable count of approximately 1 × 10^8^ colony-forming units (cfu) mL^−1^ and then diluted 1:10 in either Tris buffer or whole saliva (diluted 1:1 with deionised water) collected from the authors, prior to use in the bacterial adherence assay.

### 2.4. Preparation of Macroporous p(HEMA) Hydrogels

A series of macroporous hydrogels were prepared by free radical polymerisation of 2-(HEMA) in the presence of deionised water (40 or 60% *w*/*w*) containing a range of molar concentrations of sodium chloride (0–0.7 M) and 0.5% *w*/*w* 2,2′-azo-bis(2-methylpropionitrile) (AIBN). The resultant monomer solution was then injected into a mould comprising two glass sheets (150 mm × 100 mm, J.E. Harrison and Co., Ltd., Glaziers, Belfast, UK), containing a loop of silicone tubing (to contain the solution) (outer diameter 1.19 mm, internal diameter 0.63 mm, SF Medical, Hudson, MA, USA) and clamped to avoid leakage. The mould was placed in a vacuum oven at 60 °C for 16 h (*circa* 50 mbar) to facilitate completion of the polymerisation reaction. The polymer was then removed from the mould and submerged in deionised water for 4 weeks to remove any residual sodium chloride. All hydrogels were then stored in deionised water until required for analysis.

### 2.5. Preparation of Gentamicin Containing Macroporous p(HEMA) Hydrogels

Gentamicin loading of microporous hydrogels was performed by storage of dried microporous hydrogels in a solution of gentamicin sulphate (Tris, pH 7.4, 50 mg mL^−1^) for 48 h. Gentamicin uptake into macroporous hydrogels (following swelling) was quantified by high-performance liquid chromatography (HPLC) with precolumn derivatisation using 2,4-dinitrofluorobenzene, as previously reported [24].

### 2.6. Buffer Uptake of Macroporous p(HEMA) Hydrogels

Discs (*circa* 12 mm diameter and 4 mm thickness) were cut from washed and hydrated p(HEMA) macroporous hydrogels and dried in an oven until steady-state weight was achieved. The discs were then accurately weighed and transferred to stoppered vials containing Tris buffer (pH 7.4, 1 M) and incubated under shaking conditions (100 oscillations min^−1^) at 37 °C. After storage for 48 h, each sample was removed, blotted dry between two pieces of filter paper (to remove excess surface water), and then reweighed before being returned to the stoppered vial until the next sampling period. The buffer uptake was calculated as an equilibrium swelling ratio, namely the ratio of the mass gain at equilibrium swelling to the original mass of the dried macroporous hydrogel, as previously reported [40].

### 2.7. Mechanical Properties of Macroporous p(HEMA) Hydrogels

The mechanical properties of the macroporous hydrogels (ultimate tensile strength, elongation at break, and Young’s modulus) were determined using a Stable Micro Systems TA-TX2 texture analyser (Godalming, Surrey, UK) and a crosshead speed of 1 mm s^−1^, as previously reported [40].

### 2.8. Gentamicin Release from Macroporous p(HEMA) Hydrogels

**Figure 1:**
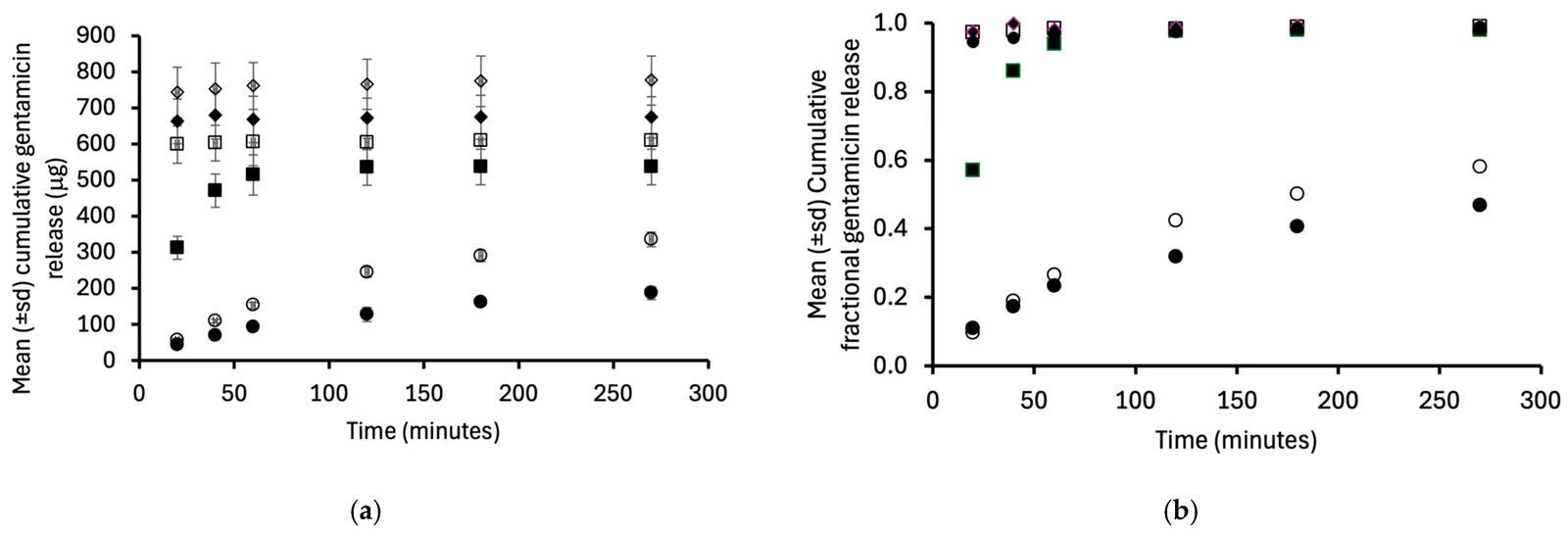
The effect of the salt concentration in the polymerisation medium on the release of gentamicin from macroporous hydrogels (40% *w*/*w* HEMA). Symbols: closed circles no NaCl, open circles 0.1 M NaCl, closed squares 0.3 M NaCl, open squares 0.5 M NaCl, closed diamonds 0.6 M NaCl, and open diamonds 0.7 M NaCl. (**a**) Mean ± sd cumulative mass of gentamicin released. (**b**) Mean fractional release of gentamicin (note standard deviations have not been included, to retain clarity; however, in all cases the coefficient of variation is less than 10%).

The release of gentamicin as a function of time from the hydrogels was assessed by immersing the samples in pre-heated Tris buffer (10 mL, pH 7.4) and placing them in a water bath under shaking conditions (100 oscillations min) at 37 °C. At selected time intervals (shown as the x axis in Figure 1), the samples were removed and immersed in fresh, pre-heated buffer. Quantification of the mass of gentamicin released at each sampling period was performed by HPLC, as detailed in Section 2.4.

### 2.9. Bacterial Adherence to Macroporous p(HEMA) Hydrogels

The hydrogel samples were placed on a needle attached to the cap of a McCartney bottle. A known volume (20 mL, *circa* 1 × 10^8^ colony-forming units mL^−1^) of each bacterial suspension (grown and treated as described in Section 2.3) was then diluted 1:10 in either Tris buffer (pH 7.4, 1 M) or whole saliva (diluted 1:1 with deionised water). These dilutions were then incubated at 37 °C for 30 min in an orbital incubator set to 100 oscillations min^−1^. The saliva-treated cells were additionally centrifuged and resuspended to the same volume in Tris buffer (pH 7.4, 1 M). Discs of each material under investigation were attached to needles (three per needle) and placed into McCartney bottles containing approximately 20 mL of Tris buffer or whole saliva (diluted 1:1 with deionised water). These were then incubated at 37 °C in an orbital incubator (100 oscillations min^−1^) for defined time periods (0.5 h, 1 h, 4 h), after which the needles containing the discs were removed, and the number of adherent bacteria was enumerated, as previously described [24,39].

### 2.10. Bacterial Adherence to Hydrogels Following Nebulisation

Bacteria (4 mL, 1 × 10^9^ cfu mL^−1^) were nebulised onto the macroporous hydrogels over a 0.5 h period using a Hudson Micro Mist^®^ small volume nebulising chamber, which was attached to a small volume nebuliser (DeVilbiss SunMist Plus^®^, Drive DeVilbiss Healthcare Limited, Yorkshire, UK), as previously described [24]. The number of adherent bacteria was determined following removal from the surface of the hydrogel, as previously described [22,24].

### 2.11. Effect of Nebulisation of Gentamicin on the Persistence of Nebulised Bacteria

Two separate investigations were performed. Firstly, bacteria were nebulised onto hydrogel samples (prepared using 0.1 M NaCl) as described in Section 2.10, after which a 4 mL volume of gentamicin (80 mg mL^−1^) was transferred into the nebuliser and nebulised onto the hydrogel surface for 20 min. Secondly, a solution of gentamicin (4 mL, 80 mg mL^−1^) was nebulised onto fresh discs of microporous p(HEMA) (prepared using 0.1 M NaCl), after which up to five separate cycles of bacteria were nebulised onto the gentamicin-containing hydrogel discs. The discs were removed, and the number of adherent/persistent bacteria enumerated as described in Section 2.10.

### 2.12. Determination of the Antimicrobial Persistence of Gentamicin-Loaded Hydrogels

*S. aureus* and *Ps. aeruginosa* were grown to the stationary growth phase as described in Section 2.3. The bacterial suspension was then centrifuged at 3000× *g* for 10 min, washed in Tris buffer (pH 7.4, 1 M), and resuspended to a bacterial count of *circa* 1 × 10^7^ cfu mL^−1^. A volume of bacterial suspension (0.5 mL) was added to molten agar (20 mL), mixed, added to a Petri dish, and allowed to cool. A hydrogel disc was placed on the solid agar, and the Petri dish was incubated at 37 °C for 18 h, after which the zone of inhibition was measured with digital callipers. The disc was then removed and transferred to a freshly seed plate and incubated as before. This process was continued until no zone of inhibition was observed, this point being defined as the period of antimicrobial persistence.

### 2.13. Statistical Analysis

The effects of the concentration of water (40% *w*/*w* or 60% *w*/*w*) and the concentration of sodium chloride on drug loading, swelling in Tris buffer, mechanical properties (ultimate tensile strength, % elongation at break, and Young’s modulus) and bacterial persistence were statistically evaluated by two-way analysis of variance (ANOVA). The effects of saliva treatment on bacterial adherence to hydrogels were determined by one-way ANOVA. The effects of gentamicin nebulisation on the viability of bacteria and the effects of bacterial nebulisation on the viability of bacteria on gentamicin containing hydrogels were determined by an unpaired Student’s *t*-test. Finally, the effects of material composition and time on gentamicin release from the macroporous hydrogels were examined by two-way repeated-measures ANOVA. Individual differences between the means of treatments were determined using Tukey’s HSD test, and a *p*-value of less than 0.05 denoted significance. In all cases, five replicate measurements were taken. All statistical tests were performed using JMP Student Edition Version 19 (JMP Statistical Discovery LLC, 920 SAS Campus Drive, Cary, NC, USA).

## 3. Results and Discussion

Ventilator-associated pneumonia (VAP) is a life-threatening condition which may occur 48 h post-endotracheal intubation and is linked to microbial colonisation on the lumen of the endotracheal tube [32,41]. As such, identifying methods to prevent or treat infection is critical. Non-pharmacological approaches have been suggested for the prevention of VAP and include minimisation of the time for ventilation and elevation of the position of the patient’s head (between 30° and 45°), thereby minimising gastric reflux and aspiration [42,43]. Given the oral cavity’s role as a portal for infection, selective decontamination of the digestive tract has been reported. To achieve this, the patient receives a cocktail of antimicrobial agents that act only within the gastrointestinal tract (due to their low gastrointestinal absorption), including tobramycin, colistin, polymyxin E, and amphotericin B, augmented by intravenous antibiotic therapy [41]. In one study, de Smet et al. reported a 3.5% decrease in mortality rate associated with SDD [44]. Similarly, Bos et al. reported that SDD decreases mortality rates [45]. One concern with this approach is the potential emergence of bacterial resistance. Other approaches include the incorporation of antimicrobial agents into the endotracheal tube, e.g., hexetidine [37], silver [46], and antibiotics [47,48], and the use of brush-like coatings on the lumen of the endotracheal tube [49]. As an alternative strategy, Adair et al. [32] nebulised high concentrations of gentamicin into the lumen of the endotracheal tube and reported that this method was more effective in reducing biofilm than parenteral cephalosporin. To reduce the high concentrations of nebulised gentamicin, we described a new concept for the prevention of VAP using hydrogel luminal coatings on endotracheal tubes that can effectively entrap nebulised antibiotics and, in so doing, provide an antimicrobial sink that reduces microbial colonisation and persistence [24]. Whilst successful in reducing or eliminating bacteria from their surface, coating of the lumen surface with the hydrogel raised significant difficulties, notably deposition of the coating (ensuring uniformity of the coating along the full length of the endotracheal tube and integrating this stage into the manufacturing process for the endotracheal tube) and maintaining attachment of the coating to the endotracheal tube base polymer (usually polyvinyl chloride). Considering this, we hereby describe a novel approach that employs macroporous hydrogels as removable liners for endotracheal tubes. When placed within the lumen following insertion of the endotracheal tube, their high porosity can rapidly and effectively entrap nebulised antibiotics and act as an antimicrobial sink that can rapidly eliminate adherent bacteria. Furthermore, should bacteria enter the space between the endotracheal tube and the outer surface of the microporous hydrogel liner, the antimicrobial solution at this interface will similarly eradiate bacteria. This approach will extend the period during which the endotracheal tube may be used (thereby obviating the need for tube removal) and reduce the incidence of VAP.

### 3.1. Mechanical Properties of Macroporous Hydrogels

Considering the importance of mechanical properties in the insertion and removal of the liner material, the macroporous hydrogels were characterised in terms of the ultimate tensile strength (UTS), % elongation at break (% Elongation), and Young’s modulus (YM), as determined by tensile analysis (Table 1).

Increasing sodium chloride concentration and the water content of the polymerisation mixture significantly decreased all three mechanical properties under investigation. To understand these observations, consideration must be given to the structure of the resultant hydrogels. Hydrogels prepared using a 40% aqueous phase were transparent, whereas those prepared using a 60% aqueous phase were opaque, heterogeneous materials [50,51]. This is attributed to the contrasting solubility between HEMA (for which water is an excellent solvent) and p(HEMA), for which there is a decrease in the water solubility. The monomer phase separates into droplets, resulting in a sponge-type structure [52]. The porosity of these materials may be further engineered by adding salt to the polymerisation medium *via* the salting-out effect [53], with a concentration range of 0.3–0.7 M reported to be most effective [52]. Scanning electron micrographs of p(HEMA) hydrogels containing 60% *w*/*w* water and a range of NaCl concentrations in the polymerisation medium are presented in Figure 1 and illustrate the increasing porosity of the materials as salt concentration increases. Thus, the mechanical properties of the macroporous hydrogels prepared using 40% HEMA monomer decrease as the porosity increases (through increased salt concentration), with the pores offering a nidus for deformation. The effects of salt concentration on the mechanical properties were greater in hydrogels prepared using 0.5–0.7 M NaCl, and this may be attributed to the interconnection of the pores within the matrix, as previously proposed [52,53,54]. Conversely, whilst salt concentration affects the mechanical properties of p(HEMA) macroporous hydrogels prepared using 40% *w*/*w* water, these effects were more modest in comparison to the hydrogels prepared using 60% *w*/*w* water. This relates to the greater heterogeneous nature of the latter material that results from the lower concentration of water in the polymerisation medium [50]. It is important to reflect on the mechanical properties and relevance to the proposed use of macroporous hydrogels as removable lining materials for endotracheal tubes. Such devices must be easily inserted and removed from the lumen of the device, and accordingly the ultimate tensile strength, % elongation at break, and Young’s modulus are relevant to performance and should be maximised. Based on this, macroporous hydrogels prepared using lower salt concentrations would be promising candidates.

### 3.2. Swelling Properties of and Gentamicin Uptake into Macroporous Hydrogels

The proposed use of the removable macroporous liner requires that, following nebulisation of gentamicin into the lumen of the endotracheal tube, it will be trapped within the hydrogel matrix through a swelling mechanism. Thus, the swelling properties and gentamicin uptake, both at equilibrium, were determined; the results are shown in Table 2. Increasing salt concentration significantly increased both the equilibrium swelling ratio and gentamicin loading; a strong correlation was observed between these two parameters. The monomer-to-water ratio also affected the equilibrium swelling ratio and gentamicin uptake; hydrogels manufactured with 40% HEMA exhibited higher equilibrium swelling ratio and gentamicin uptake than those manufactured with 60% HEMA. These observations can again be explained by referring to the macroporous structure of the hydrogels; the greater porosity associated with increasing salt and water concentrations resulting in increased swelling and gentamicin uptake [52,55].

### 3.3. Release of Gentamicin from Macroporous Hydrogels

An important attribute of candidate macroporous hydrogels is the capacity to release gentamicin in an aqueous environment. Accordingly, the release of gentamicin from macroporous hydrogels prepared with 40% HEMA monomer (60% *w*/*w* water) and various salt concentrations was studied. This series of hydrogels was selected for high capacity for gentamicin loading, as described in Section 3.2. The release properties are presented in Figure 1a,b as the cumulative mass and cumulative fractional release of gentamicin, respectively. The gentamicin loading values of macroporous hydrogels prepared using 0 M, 0.1 M, 0.3 M, 0.5 M, 0.6 M, and 0.7 M were 393 μg, 568 μg, 540 μg, 492 μg, 782.0 μg, and 681 μg, respectively. The concentration of salt in the polymerisation medium affected the release of gentamicin, though not in a sequential manner. As the salt concentration is increased from 0.0 M to 0.1 M to 0.3 M, the mass and fraction of gentamicin released as a function of time increased in a diffused-controlled fashion [56]. The masses of gentamicin released from hydrogels prepared using 0.5–0.7 M NaCl were statistically similar, and complete release occurred before or at the first sampling point (20 min). This characteristic may be accredited to the greater porosity and the interconnectivity between the pores in these materials [52,55]; these facilitated uncontrolled release of the antimicrobial agent. Normally, platforms that provide uncontrolled (burst) release would not receive attention for pharmaceutical applications; however, for this application, this is advantageous. Following adherence to the removable macroporous hydrogel liner, bacteria will be instantaneously exposed to a high concentration of gentamicin, ensuring their eradication [57]. It should be noted that the surface of the removable luminal liners will be bathed only in an aqueous film resulting from the inspiration of moist air through the lumen of the endotracheal tube, to mimic normal physiological conditions and minimise tracheal damage [58,59]. Given the restricted volume of liquid adjacent to the lumen of the endotracheal tube, the availability of a solution of gentamicin at a high concentration is advantageous and obviates the need for/challenges associated with the dissolution of antimicrobial agents within this fluid layer.

### 3.4. Determination of Antimicrobial Persistence of Macroporous Hydrogels

**Figure 2:**
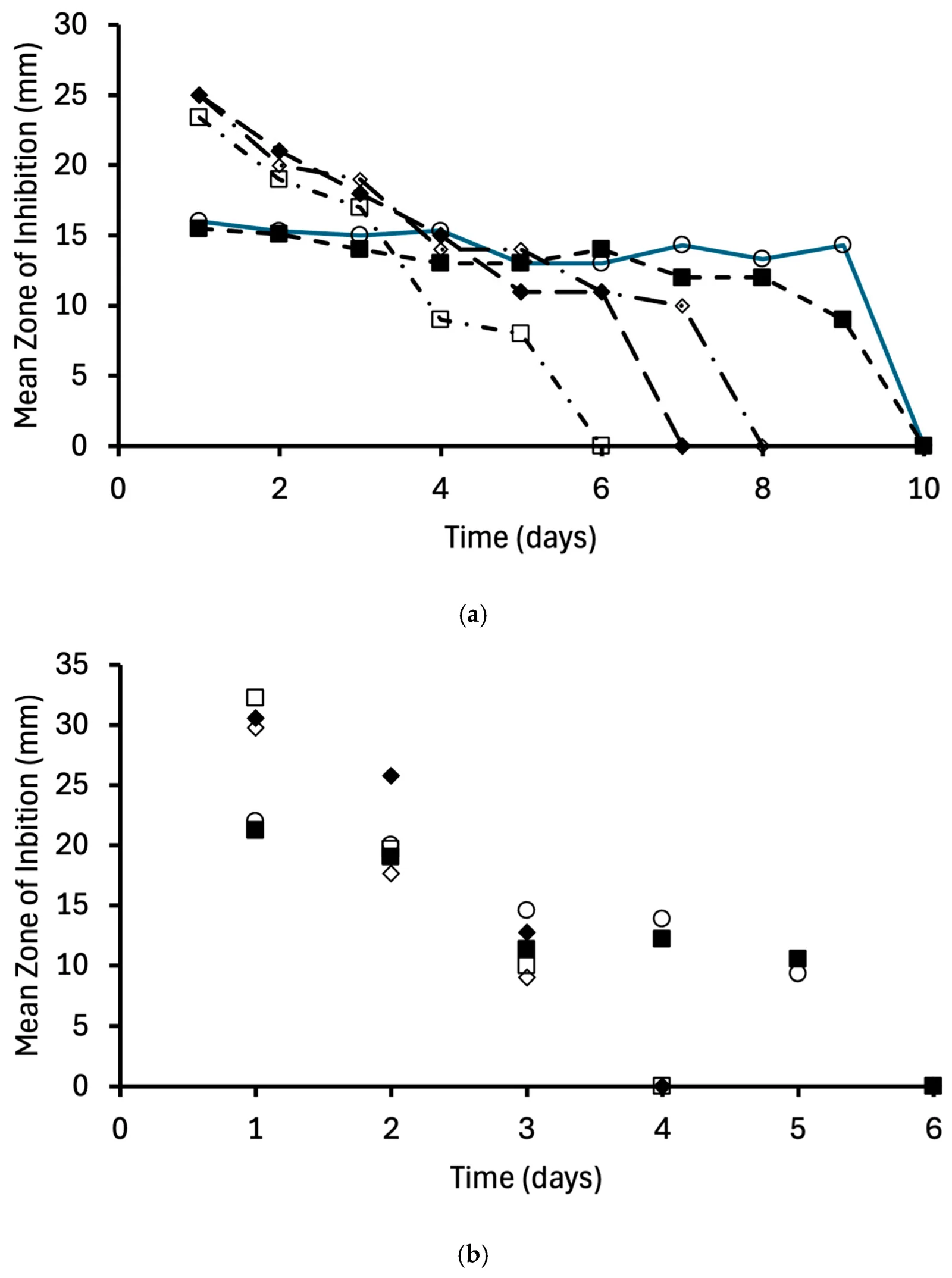
Antimicrobial persistence of macroporous p(HEMA) hydrogels (40% *w*/*w* HEMA) against *S. aureus* (**a**) and *Ps. aeruginosa* (**b**). Symbols: open circles 0.1 M NaCl, closed squares 0.3 M NaCl, open squares 0.5 M NaCl, closed diamonds 0.6 M NaCl, and open diamonds 0.7 M NaCl. Standard deviations have not been presented, to retain clarity; however, in all cases, the coefficient of variation was less than 8%.

In the clinical environment (as noted in Section 3.3), there is limited fluid volume in the endotracheal tube lumen, resultant from inspiration of air of defined relative humidity [24]. Therefore, whilst conventional drug release methodology offers information regarding the mechanism of drug release from a delivery system, it is important to consider drug release under conditions of restricted fluid volume for the drug to diffuse into. Accordingly, we have used zone-of-inhibition measurements and successive transfers of the hydrogel (prepared using 60% *w*/*w* water and different concentrations of NaCl) until no zone of inhibition was observed to assess the persistence of antimicrobial activity [24,40]. Both the magnitude of the zones of inhibition and the time to the absence of a zone of inhibition (persistence) were affected by the salt concentration in the polymerisation medium for both *S. aureus* and *Ps. aeruginosa* (Figure 2a and Figure 2b, respectively). These results may be understood with reference to the drug release data. Macroporous hydrogels prepared using 0.5–0.7 M sodium chloride released the drug rapidly, and these materials produced the greatest zones of inhibition but exhibited the lowest persistence of both microorganisms.

Conversely, macroporous hydrogels which exhibited greater control of gentamicin release displayed lower initial zones of inhibition. However, these materials displayed greater antimicrobial persistence. Importantly, these results highlight the potential of the materials to provide persistent antimicrobial activity under conditions in which the volume of fluid into which the drug can partition is restricted [24]. This offers opportunities to rationalise the number of nebulisations required during endotracheal tube use.

### 3.5. Bacterial Adherence to Macroporous p(HEMA) Hydrogels

**Figure 3:**
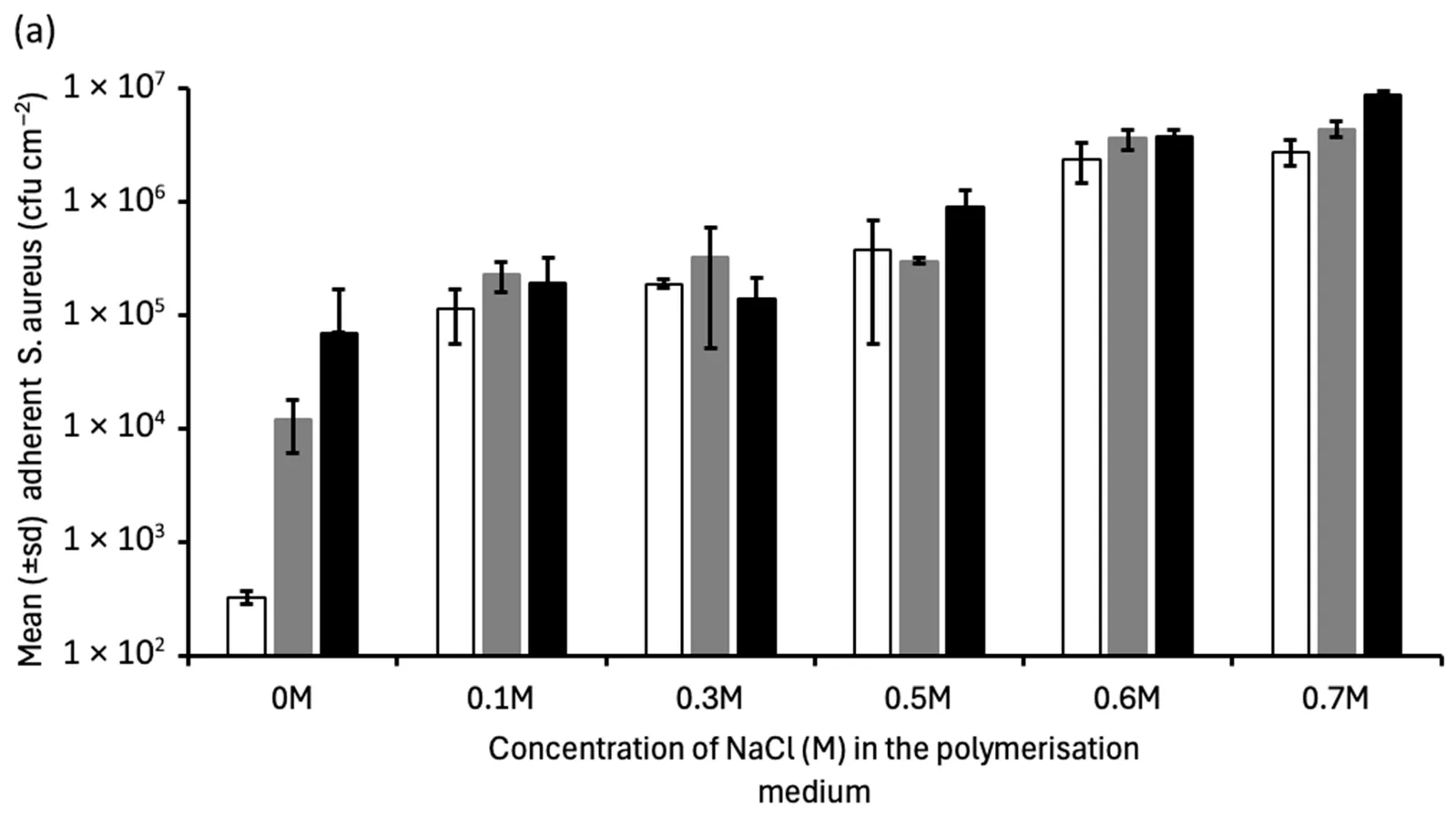

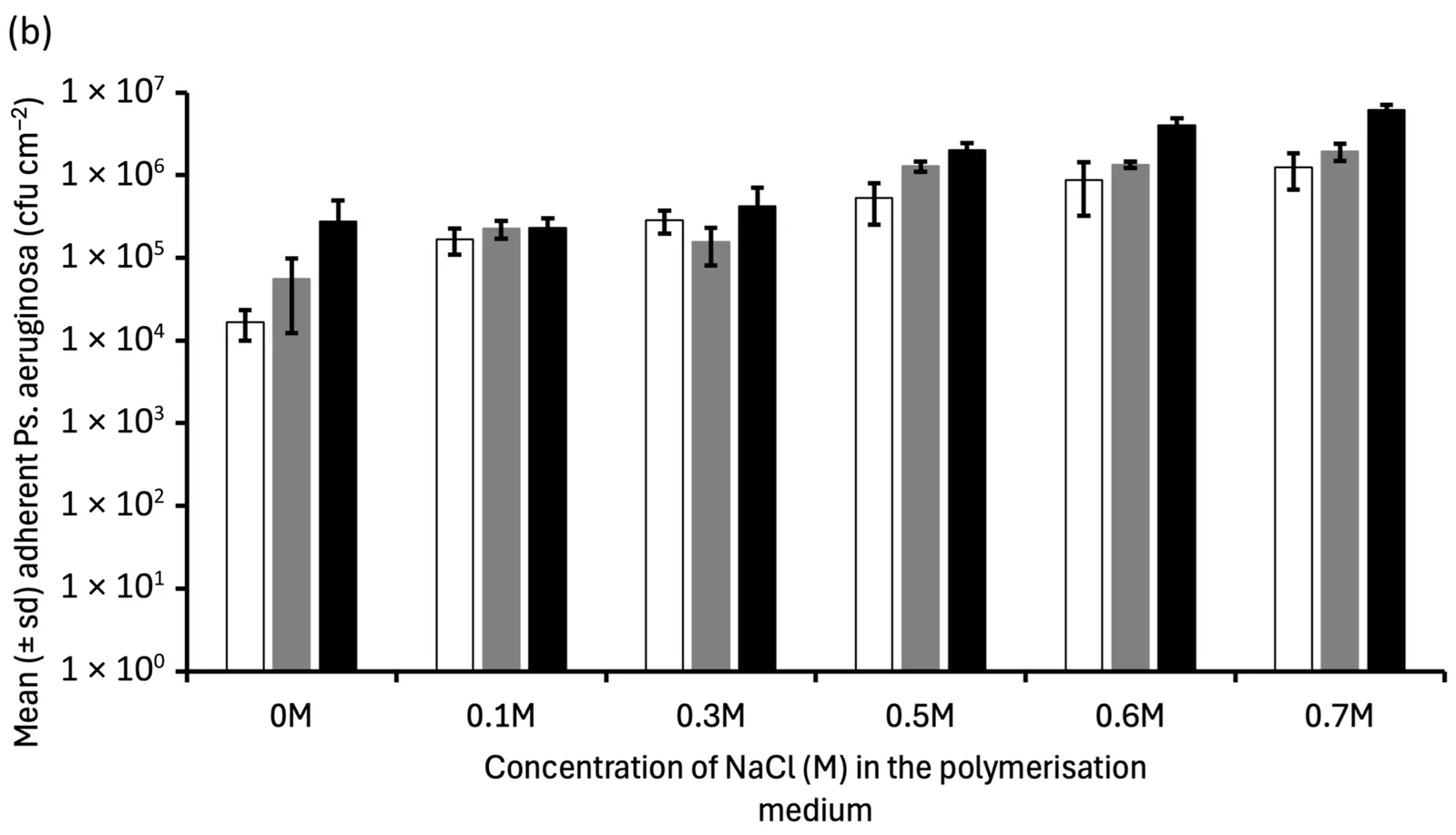
The effect of contact time on the mean (±sd) log number of adherent *S. aureus* (**a**) and *Ps. aeruginosa* (**b**) to macroporous hydrogels containing 40% *w*/*w* HEMA and prepared using a range of salt concentrations in the polymerisation medium. Symbol: clear bars represent a 0.5 h contact time, grey bars represent a 1 h contact time, and black bars represent a 4 h contact time.

The adherence of microorganisms is considered the first stage in the colonisation of medical devices, which can ultimately lead to device-related infections, i.e., VAP [60,61]. Adherent bacteria progress to form a biofilm and, in so doing, exhibit increased resistance (up to 1000×) to antimicrobial agents and the body’s immune system [62]. As medical device surface porosity increases, bacterial adherence and biofilm formation have been reported to increase [63]. For example, Braem et al. studied the colonisation of porous titanium coatings for orthopaedic implants and showed that reducing porosity reduced bacterial colonisation [64]. Similarly, the greater bacterial adherence to mesoporous bioceramics was attributed to their porosity [65]. Initially, this study investigated the effect of salt concentration in the polymerisation medium (and hence hydrogel porosity) on the subsequent adherence of *S. aureus* and *Ps. Aeruginosa* (Figure 3a and Figure 3b, respectively).

Increasing the contact time between the microorganisms and the macroporous p(HEMA) and increasing the salt concentration in the polymerisation medium significantly increased the mean number of adherent bacteria on the macroporous hydrogels. As previously noted, the porosity of the macroporous hydrogels increased as the concentration of salt in the polymerisation medium increased (Section 3.1). Therefore, the increasing adherence of the two isolates to macroporous p(HEMA) with increasing NaCl concentration may be attributed to increased hydrogel porosity, reiterating the contribution of biomaterial porosity to microbial adherence, as previously reported [66,67,68].

Given the possible role of biological fluids in the adherence of microorganisms to medical devices [69,70], the effect of saliva on the adherence of *S. aureus* and *Ps. aeruginosa* was examined (Table 3). Biological fluids, e.g., saliva, have been reported to deposit a conditioning film on the surface of medical devices to which bacteria may adhere [71]. The ability of saliva to modify biofilm growth and structure has been reported, e.g., Choi et al. [72] and Inui et al. [73]. In this study, there was no discernible effect of saliva coating of the bacteria and/or macroporous hydrogels on subsequent microbial adherence; however, the intra-group variability may have masked certain effects.

### 3.6. Effect of Gentamicin Nebulisation on Bacterial Persistence on Macroporous p(HEMA) Hydrogels

Using our previous nebulisation model [24], the effect of nebulisation of gentamicin solution on the persistence of previously nebulised *S. aureus* and *Ps. aeruginosa* on macroporous p(HEMA), prepared using 0.1 M NaCl in the polymerisation medium, was determined. The adherence densities of *S. aureus* and *Ps. aeruginosa* on microporous p(HEMA) were 6.3 ± 1.8 × 10^5^ cfu cm^−2^ and 3.8 ± 1.2 × 10^4^ cfu cm^−2^, respectively. Following nebulisation of a gentamicin solution, no viable organisms of either species were detected, highlighting the efficacy in entrapping gentamicin and eradicating adherent bacteria. In the second experimental scenario, several cycles (less than or equal to five) of bacteria were nebulised onto macroporous p(HEMA) that had previously been exposed to a nebulised gentamicin solution. After each cycle, the number of viable bacteria associated with the hydrogel was enumerated. Notably, no viable bacteria of each species were detected, regardless of the number of cycles of nebulised bacteria to which the material had been exposed. This illustrates the antimicrobial resilience of the macroporous removable liner materials and suggests that less frequent nebulisation of gentamicin may be clinically appropriate.

## 4. Conclusions

In this study, we described the formulation and characterisation of macroporous p(HEMA) hydrogels designed as removable liners for endotracheal tubes to reduce the incidence of ventilator-associated pneumonia. The macroporous hydrogels were prepared by free-radical polymerisation incorporating a range of sodium chloride concentrations (0.1–0.7 M) and either 40% *w*/*w* or 60% *w*/*w* water in the reaction medium. Increasing the concentration of salt significantly increased the porosity and reduced the ultimate tensile strength, % elongation at break, and Young’s modulus of the hydrogels. The mechanical properties of hydrogels prepared with 60% *w*/*w* water (40% *w*/*w* monomer) were more compromised than those of hydrogels prepared with 40% *w*/*w* water (60% *w*/*w* monomer). Increasing the salt content within the polymerisation medium significantly increased the equilibrium swelling ratio of and gentamicin uptake into macroporous hydrogels prepared using 60% *w*/*w* water (40% *w*/*w* monomer) and may be attributed to the increased porosity of these materials. Conversely, the equilibrium swelling and gentamicin uptake of macroporous hydrogels prepared with 40% *w*/*w* water (60% *w*/*w* monomer) were independent of salt concentration and were statistically lower than those of hydrogels prepared with 60% *w*/*w* water (40% *w*/*w* monomer). To balance mechanical properties (to facilitate insertion and removal of the removable liner) and gentamicin uptake, macroporous hydrogels prepared with 60% *w*/*w* water (40% *w*/*w* monomer) were selected for further study. Gentamicin release from macroporous p(HEMA) was dependent on the concentration of salt in the polymerisation medium. Gentamicin release from hydrogels prepared using 0.1 M and 0.3 M NaCl was controlled, whereas release from hydrogels prepared with higher NaCl concentrations was rapid, typically with ≥90% of the drug released at the first sampling point (20 min). These observations were attributed to the increased porosity of the hydrogels prepared using higher salt concentrations. The persistence of antimicrobial activity was assessed by measuring zones of inhibition against *S. aureus* and *Ps. aeruginosa* after repeated passages of the macroporous hydrogels. The initial zones of inhibition for hydrogels prepared using higher concentrations of NaCl (e.g., 0.6 M and 0.7 M) were greater than those for hydrogels prepared using lower concentrations of NaCl and may be linked to the faster release of gentamicin. Conversely, the persistence of antimicrobial activity was greater for hydrogels prepared using lower concentrations of NaCl and relates to the controlled release of gentamicin from these hydrogels. Bacterial adherence, the initial stage in biofilm formation on medical devices, to macroporous p(HEMA), prepared using 60% *w*/*w* water (40% *w*/*w* monomer) and a range of salt concentrations, was studied. The adherence of *S. aureus* and *Ps. aeruginosa* to macroporous hydrogels increased with increasing salt concentration used to prepare the hydrogels and can again be attributed to the increasing porosity. Given the need to insert and remove hydrogel liners from endotracheal tubes to facilitate high gentamicin loading and availability to eradicate pathogens and to minimise microbial adherence, the macroporous hydrogels prepared at lower NaCl concentrations were deemed more clinically appropriate. Accordingly, macroporous hydrogels prepared using 0.1 M NaCl were further investigated in a nebulisation model based on clinical practice, as previously described by the authors. Initially, the effects of saliva pretreatment of the bacterial species and/or the macroporous hydrogel were examined and shown not to affect microbial adherence. Following nebulisation of bacterial suspensions, both *S. aureus* and *Ps. aeruginosa* adhered to the macroporous hydrogel. Subsequent nebulisation of a gentamicin solution eradicated adherent microorganisms. In a related experiment, gentamicin was initially nebulised on macroporous p(HEMA), and then up to five additional passages of bacterial suspension (*S. aureus* or *Ps. aeruginosa*) were nebulised onto the hydrogel sample, and their viability was determined. As before, no viable microorganisms were recovered from the macroporous hydrogel. Therefore, it is proposed that this macroporous hydrogel (manufactured in the presence of 60% *w*/*w* water, 40% *w*/*w* monomer, and 0.1 M NaCl) offers the correct balance of mechanical and antimicrobial properties to work effectively as a removable liner for endotracheal tubes. It is suggested that the approach described herein represents a paradigm shift in preventing ventilator-associated pneumonia associated with endotracheal tubes. Future studies are required to assess the performance of the hydrogel liners in simulated in-use environments. These studies should include, but not be limited to, confirming retention of the liner within the endotracheal tube under airflow conditions; characterising airflow dynamics within endotracheal tubes containing the liner; evaluating the processes of liner insertion and removal; and understanding and controlling antibiotic solution entrapment along the full length of the liner. Optimisation of the formulation and use of the liner hydrogel will ensure that, given the limited drug loss from the liner (in the absence of conventional drug dissolution with the intubated respiratory system), the optimal antimicrobial therapy would require a minimum number of nebulisation cycles. In so doing this will minimise the use of gentamicin and the possible emergence of antimicrobial resistance.

## Figures and Tables

**Table 1:** The effect of hydrogel composition on the mean (±sd) ultimate tensile strength (UTS), Young’s modulus (YM), and % elongation at break (% Elongation).

HEMA:Water	NaCl Conc^n^ (M)	UTS (MPa)	YM (MPa)	% Elongation
40:60	0.1	0.43 ± 0.08	0.65 ± 0.03	105.60 ± 22.80
	0.3	0.33 ± 0.04	0.60 ± 0.03	81.70 ± 12.40
	0.5	0.07 ± 0.01	0.23 ± 0.02	58.89 ± 4.88
	0.6	0.05 ± 0.01	0.21 ± 0.05	37.38 ± 6.59
	0.7	0.04 ± 0.00	0.25 ± 0.04	23.30 ± 8.64
60:40	0.1	0.64 ± 0.07	1.10 ± 0.03	135.20 ± 18.74
	0.3	0.68 ± 0.03	1.09 ± 0.10	107.20 ± 13.90
	0.5	0.73 ± 0.12	1.16 ± 0.04	115.88 ± 13.04
	0.6	0.42 ± 0.03	1.14 ± 0.01	51.37 ± 3.46
	0.7	0.42 ± 0.07	1.08 ± 0.05	54.22 ± 11.54

**Table 2:** The effect of hydrogel composition on the mean (±sd) swelling ratio and gentamicin loading at equilibrium.

HEMA:Water	NaCl Conc^n^ (M)	Swelling Ratio	Gentamicin Loading (μg cm^−2^)
40:60	0.1	1.23 ± 0.03	570.0 ± 34.3
	0.3	1.21 ± 0.02	541.4 ± 49.4
	0.5	1.57 ± 0.01	615.2 ± 40.9
	0.6	1.93 ± 0.07	680.1 ± 53.1
	0.7	1.86 ± 0.11	785.8 ± 60.2
60:40	0.1	0.61 ± 0.00	85.2 ± 5.0
	0.3	0.62 ± 0.00	91.4 ± 7.5
	0.5	0.70 ± 0.01	91.1 ± 5.8
	0.6	0.78 ± 0.03	98.9 ± 4.3
	0.7	0.69 ± 0.01	95.1 ± 3.8

As removable liners, ideally the uptake of gentamicin should be maximised, and thus this would render the hydrogels prepared using 60% HEMA monomer as potentially unsuitable for the chosen application.

**Table 3:** The effects of pretreatment of bacteria and/or macroporous p(HEMA) * on the subsequent adherence of *S. aureus* and *Ps. aeruginosa*.

Bacterial Treatment	Biomaterial Treatment	Mean (±sd) Adherence (cfu cm^−2^ × 10^5^)	
		*S. aureus*	*Ps. aeruginosa*
Tris buffer	Tris buffer	1.11 ± 0.30	2.05 ± 0.92
	Pooled saliva	2.38 ± 0.89	1.61 ± 0.55
Pooled saliva	Tris buffer	2.55 ± 0.80	1.52 ± 0.61
	Pooled saliva	4.03 ± 1.80	2.16 ± 0.34

* Prepared using 0.3 M NaCl, bacterial contact time 4 h.

## Data Availability

The data presented in this study are available on request from the corresponding author due to commercial sensitivity.
